# Applications of AOTF Spectrometers in In Situ Lunar Measurements

**DOI:** 10.3390/ma14133454

**Published:** 2021-06-22

**Authors:** Jinning Li, Yuhua Gui, Rui Xu, Zehong Zhang, Wei Liu, Gang Lv, Meizhu Wang, Chunlai Li, Zhiping He

**Affiliations:** 1 Key Laboratory of Space Active Opto-Electronics Technology, Shanghai Institute of Technical Physics of the Chinese Academy of Sciences, Shanghai 200083, China; lijinning@mail.sitp.ac.cn (J.L.); yhgui@mail.ustc.edu.cn (Y.G.); xurui@mail.sitp.ac.cn (R.X.); lvgang@mail.sitp.ac.cn (G.L.); wangmeizhu2013@163.com (M.W.); 2 University of Chinese Academy of Sciences, Beijing 100049, China; 3 No.26 Research Institutes of China Electronics Technology Group Corporation, Chongqing 400060, China; 13637790568@163.com (Z.Z.); liuwei@163.com (W.L.)

**Keywords:** AOTF spectrometer, in situ measurement, lunar surface

## Abstract

Spectrometers based on acousto-optic tunable filters (AOTFs) have several advantages, such as stable temperature adaptability, no moving parts, and wavelength selection through electrical modulation, compared with the traditional grating and Fourier transform spectrometers. Therefore, AOTF spectrometers can realize stable in situ measurement on the lunar surface under wide temperature ranges and low light environments. AOTF imaging spectrometers were first employed for in situ measurement of the lunar surface in the Chinese Chang’e project. The visible and near-infrared imaging spectrometer and the lunar mineralogical spectrometer have been successfully deployed on board the Chang’e-3/4 and Chang’e-5 missions. In this review, we investigate the performance indicators, structural design, selected AOTF performance parameters, data acquisition of the three lunar in situ spectral instruments used in the Chang’e missions. In addition, we also show the scientific achievement of lunar technology based on in situ spectral data.

## 1. Introduction

Compared to remote sensing spectral measurement and post-sampling laboratory spectral measurement, in situ spectral measurement allows one to investigate targets at close range with millimeter resolution, without destroying the original state of the target. This leads to intriguing possibilities for studying surface topography and material composition [1]. However, in situ measurement is challenging for spectroscopic instruments. Especially, deep space exploration requires instruments to obtain high-quality and reliable spectral data under extremely complex environmental conditions [2].

The acousto-optic tunable filter (AOTF) is an electronically tunable dispersive optical device without any moving parts. It can change the wavelength of the output diffracted light by controlling the input radio frequency (RF) [3]. Harris and Wallace first proposed a collinear AOTF in 1969 [4]. In 1974, Chang et al. used TeO_2_ to construct a noncollinear AOTF [5], which overcame the drawbacks of the collinear AOTF in terms of limited crystal availability and complicated design. In 1987, an AOTF spectrometer was used for ocean observations in the Soviet satellite “Ocean-O1-N2” [6]. In 2003, the SPICAM instrument onboard the European Space Agency’s Mars Express mission realized the first deep space application of AOTF for Martian atmospheric analysis [7]. Since then, several AOTF instruments have been used in deep-space missions [8,9,10,11,12].

Spectrometers and imaging spectrometers using acousto-optic tunable filters have many advantages, such as small size, light weight, absence of moving parts, optional wavelength, high environmental adaptability and high spectral and spatial resolution. [13,14,15] Therefore, it is suitable for the in situ spectral and imaging measurement of extraterrestrial bodies. The Chinese Chang’e Project has implemented three AOTF spectrometers for in situ spectral measurement of the lunar surface: the visible and near-infrared imaging spectrometers (VNIS) onboard Chang’e-3 (2013) [16] and Chang’e-4 (2018) [17] unmanned lunar rovers and the lunar mineralogical spectrometer (LMS) onboard Chang’E-5 (2020) [18]. VNIS onboard Chang’e-3 was the first instrument to realize in situ imaging spectral measurement of the lunar surface. The MicrOmega onboard the Japanese Hayabusa-2 (2014) mission realized the in situ spectral measurement of asteroids [19,20]. The SuperCam mounted onboard NASA’s MARS 2020 Perseverance rover is also equipped with an infrared in situ spectra submodule to realize in situ spectra of the Martian surface [21,22,23]. In addition, the ExoMars scheduled for launch in 2022 will also be equipped with an in situ spectral instrument, ISEM [24,25], to perform in situ spectral surveys of the Martian surface.

In this review, we report the design, performance specifications, and performance test results of the three AOTF spectrometers used for lunar in situ measurement in the Chang’e mission. We also report the material, tuning relationship and resolution of AOTFs used on these three AOTF in situ spectrometers. In addition, the effectiveness and results of the AOTF spectrometers are briefly described.

## 2. Lunar In Situ Spectrometers

The Yutu lunar rover in the Chang’e-3 mission carried the VNIS, which was the first AOTF hyperspectral imager to realize in situ measurement in deep space [16,26,27,28,29]. The main objectives of the mission are to perform visible and near-infrared spectral imaging (400–900 nm) and short-wave infrared spectral measurements (900–2400 nm) of the lunar surface targets. The VNIS can obtain spectral and geometric imaging data of objects on the lunar surface. The mission also focused on accomplishing in situ analyses of the mineral composition, content (abundance), and chemical composition of probe sites in the patrol area [30]. In 2018, Chang’e-4 achieved the first soft landing and roving survey on the far side of the Moon [31], and the Yutu-2 unmanned lunar rover also carried the VNIS instrument. Compared to the VNIS of the Chang’e 3 mission, the VNIS of the Chang’e 4 mission has optimized software and data acquisition logic with the addition of options for background light acquisition and subtraction [32]. The spectral resolution and the signal-to-noise ratio (SNR) were also improved. A specific performance comparison of the instruments is listed in Table 1.

In 2020, Chang’e-5 (China Lunar Exploration Program Phase III) realized China’s first lunar sampling return mission [18]. LMS was one of the key scientific payloads for Chang’e-5. LMS is inherited from the previous generation of VNIS [27]. Compared with VNIS, the spectral range of LMS was extended to 3200 nm and the SNR was also improved, as shown in Table 1. Benefitting from the improvement of spectral range and SNR, LMS can not only analyze the mineral composition, but also explore OH/H_2_O of lunar regolith.

### 2.1. VNIS on Board Chang’e-3 and Chang’e-4

**Figure 1:**
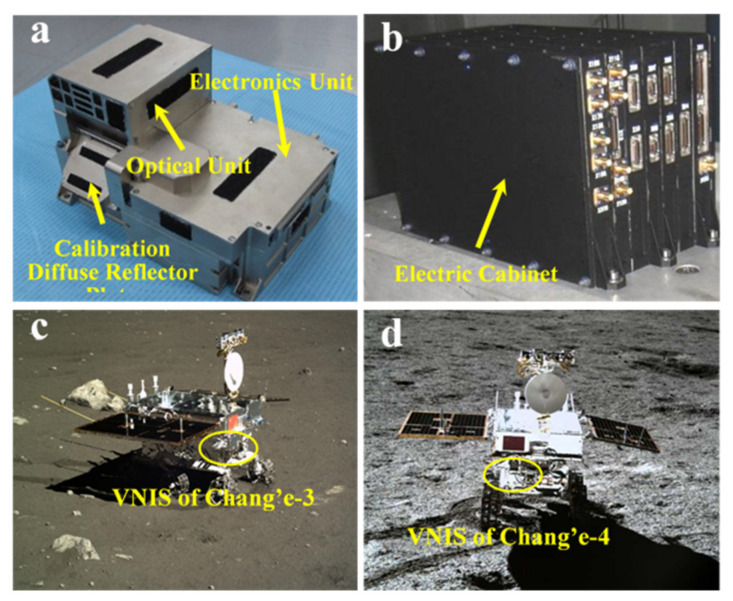
The probe composition of VNIS (**a**) and its electric cabinet (**b**); installation location of VNIS on the rover for the Chang’e-3 (**c**) and Chang’e-4 (**d**) missions.

VNIS is comprised of a measurement head and an electric cabinet, as shown in Figure 1, where the probe is installed outside the patrol cabin and the electric cabinet is installed inside the patrol cabin. The probe consists of a calibration diffuse reflector plate, an optical unit, and a probe electronics unit. The probe electronics unit includes the radio frequency (RF) driver unit, signal acquisition unit, and the main control circuit, comprised of several parts, such as the RF power amplifier, spectral measurement control, secondary power supply, and the load electric cabinet common unit.

VNIS contains two measurement channels, the visible and near-infrared (VIS-NIR 480–950 nm) and the shortwave infrared (SWIR 900–2400 nm). VIS-NIR is the imaging channel, and SWIR is the spectral acquisition channel. The VNIS on board Chang’e-3 has a spectral resolution of 2–7 nm in the VIS-NIR band and 3–12 nm in the SWIR band [29]. The VNIS on board Chang’e-4 has an improved spectral resolution of 2.4–6.5 nm in the VIS-NIR band and 3.6–9.5 nm in the SWIR band. The two channels have a field of view (FOV) of 8.5 × 8.5° and ø3.6, the measurement distance is 0.7 to 1.3 m, and the instrument can observe targets within 0.2 m^2^.

### 2.2. LMS on Board Chang’e-5

**Figure 2:**
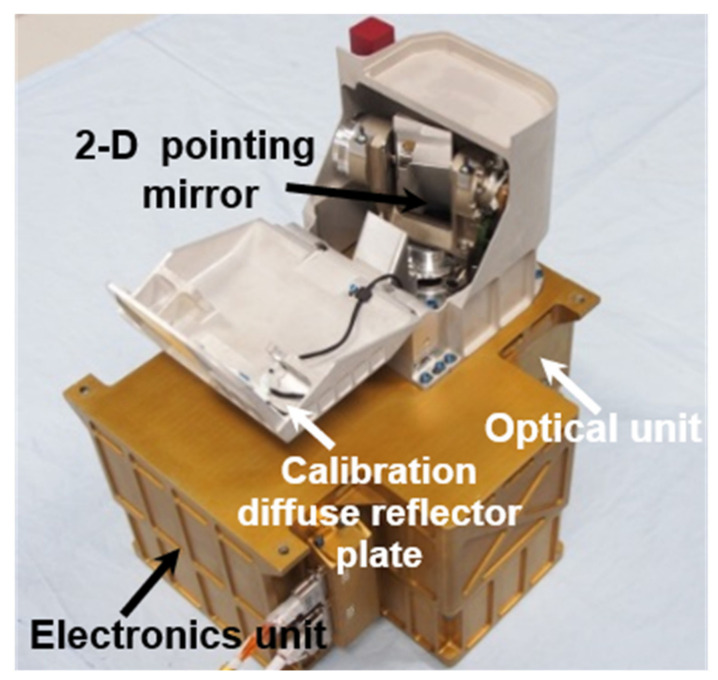
The composition of LMS.

LMS is comprised of a mounting base, optical unit, electronics unit, 2D pointing mechanism, and the dust-proofing and calibration units fitted inside the Chang’e-5 lander’s −Y + Z inclined side plate. The inclined side plate is perforated with a dust-proof device and a 2D pointing mechanism protruding from the perforations. The AOTF is encapsulated in an optical unit, which is isolated from the electronics unit for thermal control. The principle of operation is similar to that of VNIS, with the addition of a 2D pointing mechanism for selective measurement of the sampling area. The calibration plate was embedded in a dust-proof unit to realize in-flight calibration. An image and the installation location of LMS are shown in Figure 2.

LMS contains four measurement channels: the visible (VIS 480–950 nm), the near-infrared (NIR 900–1450 nm), the short-wave infrared (SWIR 1400–2300 nm), and the medium-wave infrared (MWIR 2200–3200 nm) [18]. Except for the VIS channel, which is imaged by complementary metal oxide semiconductor (CMOS), the other three channels (collectively referred to as IR) are the spectral acquisition channels. The infrared unit detectors were developed by the Shanghai Institute of Technical Physics, including InGaAs detectors for the NIR and SWIR channels, and the HgCdTe detectors for the MWIR channels. Both channels have a FOV of 4.17 × 4.17°, and can observe over a distance of 1.65 m. The VIS channel has a spectral resolution of 2.4–9.4 nm with an SNR of >34 dB, and the IR channel has a spectral resolution of 7.6–24.9 nm with an SNR of >39 dB. The pointing resolution of the 2D pointing mechanism is greater than 0.2°.

## 3. Main Characteristics of the AOTFs in VNIS and LMS

**Figure 3:**
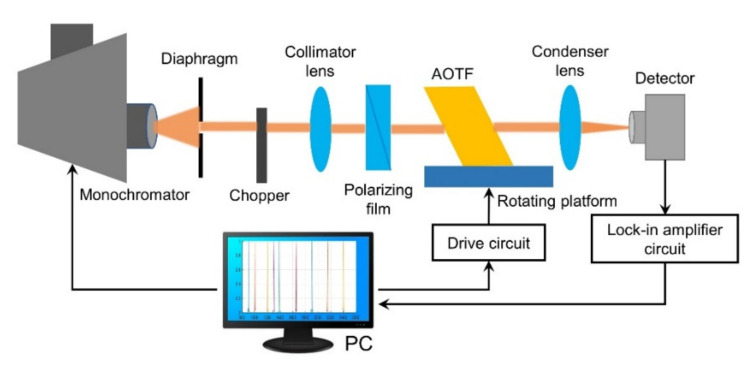
The AOTF testing system.

The performance of the AOTF significantly affects the quality of the spectrometer’s spectral characteristics. Therefore, testing the performance indicators of AOTF and investigating its ability to adapt to complex conditions on the lunar surface is the key to ensure that spectrometers obtain high-quality data. Based on the spectroscopic principle of the AOTF device, the testing scheme shown in Figure 3 was designed to test the performance indicators of six AOTFs corresponding to the VNIS on Chang’e-3 and Chang’e-4 and the LMS on Chang’e-5.

The monochromator produces narrow line-width light, which passes through a chopper to form an optical flux of variable intensity. The chopper also provides a reference signal to the lock-in amplifier circuit. A polarizing film is used in the optical path as a polarizing device to set the linear polarization direction of the light source incident on the crystal surface. By adjusting the size of the diaphragm aperture, collimated monochromatic light fills the effective aperture of the AOTF crystal to be measured. The crystal was placed on a rotating platform, and the RF drive signal was provided by a computer-controlled AOTF drive source. After the optical signal passes through the driven AOTF crystal, it is separated into the undiffracted 0-order light in the alternating state and the diffracted 1-order light in the corresponding polarization state. Both 0-order light and 1-order light can be used for measurement. This is because of the 0-order light measurement is easy to achieve optical alignment and improve measurement efficiency. The system measures the 0-order light. After passing through a condenser lens, the undiffracted 0-order light can be focused on the focal plane, and a photodetector is used to receive the undiffracted 0-order light. The detector converts the transformed variable-intensity optical flux into an alternating current (AC), which is amplified by the preamplifier, and then input to the lock-in amplifier. The amplified electrical signal is displayed on a computer. The main indicators include the tuning relationship between the diffraction wavelength and drive frequency, the relationship between the diffraction wavelength and diffraction efficiency, and the relationship between the diffraction wavelength and spectral resolution, as shown in Table 2.

### 3.1. AOTFs on Chang’e-3/4 and Their Indicators

**Figure 4:**
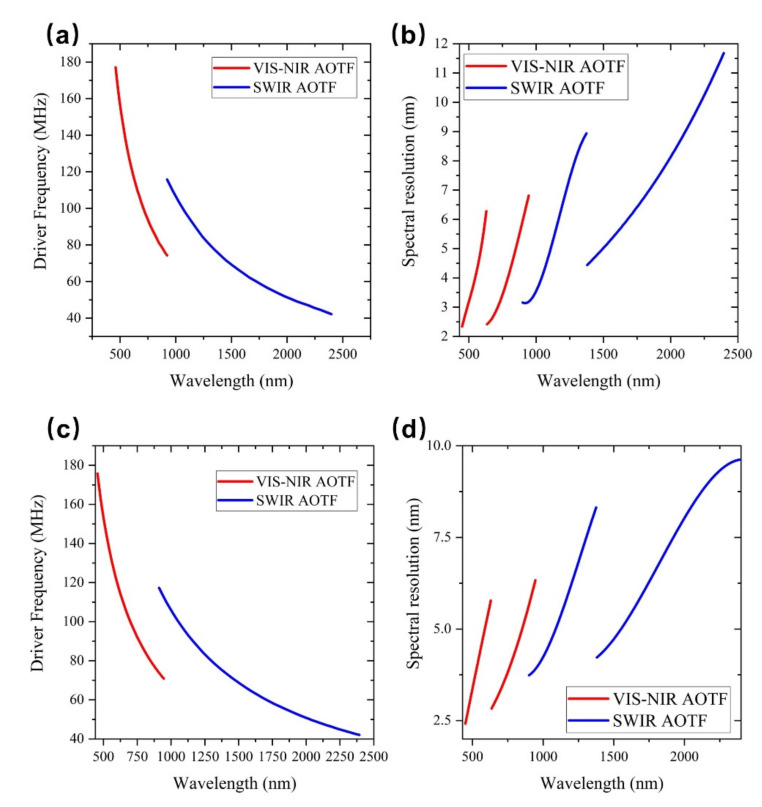
Driving frequency tuning curves of the acousto-optic tunable filters of the visible and near infrared imaging spectrometers onboard the Chang’e-3 (**a**) and Chang’e-4 (**c**); spectral resolution of the acousto-optic tunable filters vs wavelength for the Chang’e-3 (**b**) and Chang’e-4 (**d**) VNIS spectrometers.

VNIS spectrometers onboard Chang’e-3 and Chang’e-4 have two spectral measurement channels: VIS-NIR and SWIR. Each channel uses one AOTF crystal, and the two instruments use a total of four AOTF crystals. Four AOTFs were made of TeO_2_ material, and the performance indicators of four AOTFs were shown in Table 2, which were tested using the above testing system. Figure 4 shows AOTFs tuning relationship between the wavelength and the driving frequency and the relationship with the spectral resolution which used in VNIS spectrometers of Chang’e-3 and Chang’e-4.

**Figure 5:**
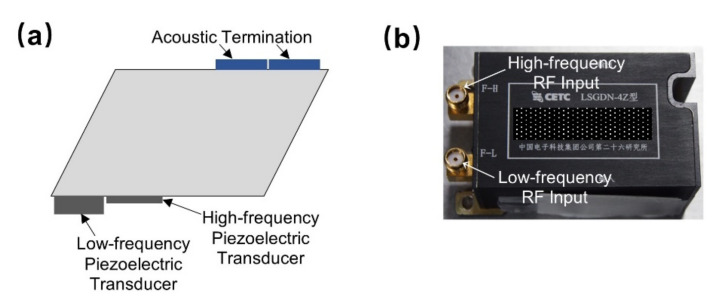
AOTF used on VNIS. (**a**) Main structure of the AOTF; (**b**) The physical object of AOTF.

AOTFs indicators used by the Chang’e-3 VNIS and the Chang’e-4 VNIS are similar, so here, we mainly discuss the AOTF used by the Chang’e-3 VNIS. The AOTF used for the VIS-NIR channel can achieve monochromatic light output from 449 to 950 nm with RF driving frequency varies from 70.7 to 178.6 MHz. The spectral resolution of the output monochromatic light varies with wavelength, and there is a boundary at 630 nm. Before 630 nm, the spectrometer resolution increased from 2.3 to 6.3 nm, and after 630 nm, the spectral resolution increased from 2.4 to 6.8 nm. The reason that the resolution is demarcated at 630 nm is due to the limited bandwidth of a single piezoelectric transducer. In order to improve the working bandwidth of AOTF, two different transducers of high frequency and low frequency are made to the switching of the two piezoelectric transducers, as shown in Figure 5. Due to the switching of the two piezoelectric transducers, the spectral resolution has a boundary at 630 nm.

For the AOTF used in the SWIR channel, the RF driving frequency changes from 41.9 to 118.9 MHz, and the spectral range varies from 899 to 2402 nm. The spectral resolution has a boundary at 1380 nm. When the spectral range is less than 1380 nm, the spectral resolution will vary from 3.1 to 8.9 nm. When the spectral range is greater than 1380 nm, the spectral resolution will vary from 4.4 to 11.6 nm.

### 3.2. AOTFs on Chang’e-5 and Their Indicators

**Figure 6:**
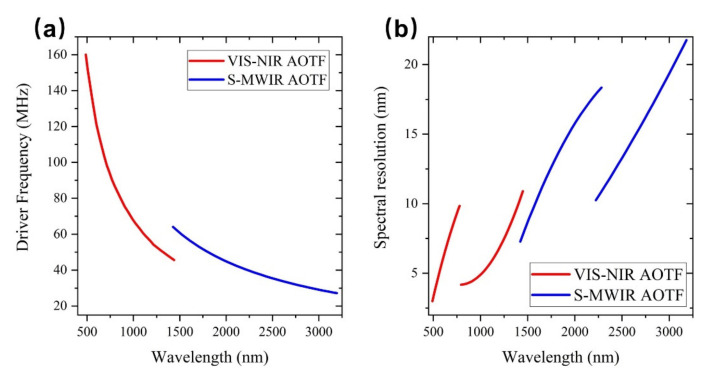
(**a**) The wavelength-frequency tuning curve of the AOTFs in the Chang’e-5 LMS; (**b**) Spectral resolution of the acousto-optic tunable filters vs. wavelength for the Chang’e-5 LMS.

Compared to the VNIS spectrometer, the LMS onboard Chang’e-5 has an extended measurement band of 3200 nm, with two channels in the VIS-NIR and SWIR-MWIR, and each channel uses one AOTF crystal. The wavelength-frequency tuning curves and spectral resolution distributions of the two AOTFs are shown in Figure 6. The AOTF used in the VIS-NIR channel can be driven by a RF of 45.2 to 163.6 MHz to obtain monochromatic light from 480 to 1450 nm. The spectral resolution is at 780 nm as the boundary, which ranges from 2.6 to 9.4 nm before 780 nm and from 2.4 to 9.0 nm after 780 nm. The RF of the SWIR-MWIR channel is 27.7 to 66.2 MHz, and the spectral range is from 1400 to 3200 nm. The spectral resolution ranges from 7.6 to 20.8 nm before 1400 nm and from 11.6 to 24.9 nm after 1400 nm.

## 4. Application Results of Lunar In situ Spectrometers

The VNIS onboard Chang’e-3 and 4 and the LMS onboard Chang’e-5 have success-fully implemented in situ measurement applications on the lunar surface, and obtained in situ imaging and spectral data with millimeter-level resolution. The scientific data obtained present a unique perspective for research on the mineral composition of the lunar surface, weathering in space, and the origin and evolution of the moon. In this section, we focus on the data obtained by the three in situ spectrometers and the corresponding scientific research reports.

### 4.1. Application Results of VNIS on Board Chang’e-3

Chang’e-3 successfully landed on the northeastern Mare Imbrium on 14 December 2013. The VNIS was booted up for the first time on 23 December 2013, and images and spectral data of the lunar soil in different regions were collected [16]. The images and spectral data acquired by the VNIS on Chang’e-3 can be used to investigate the mineral composition and chemical content of the lunar surface. Various scientific results have been published by analyzing and processing these data [33,34,35,36,37,38,39,40]. These results not only confirm the results of previous remote sensing studies, but also conclusively establish that the mineral composition of olivine tends towards iron-rich mineral end members.

Lin et al. used the modified Gaussian model (MGM) method to extract the mineralogical modal composition of lunar soil from VNIS spectral data [34]. Investigations on the mineral and chemical composition have revealed that the lunar soil was flooded with lava from volcanic eruptions approximately 2.5 billion years ago.

### 4.2. Application Results of VNIS on Board Chang’e-4

Chang’e-4 landed in the Von Kármán crater on the far side of the Moon on 3 January 2019, and commenced its scientific patrol and exploration mission. On the next day, the VNIS onboard Chang’e-4 booted up and obtained high-quality spectral data successfully from multiple test points in the landing area. The Chang’e-4 and Yutu-2 lunar rovers ended the lunar night hibernation at 21:43 and 3:54 on 6 April 2021, and entered the 29th lunar day. Until 6 April, the Yutu-2 lunar rover traveled a cumulative distance of approximately 682.8 m.

Based on the unique in situ spectral data obtained by VNIS, we have gained more information about the origin and evolution of the moon and space weathering [1,31,41,42,43,44,45,46,47,48,49,50,51,52,53]. Li et al. further analyzed the REFF spectra collected by VNIS on the first lunar day. They demonstrated that a deep medium dominated by olivine and low-calcite pyroxene exists in the south pole-Aitken basin on the far side of the moon by analyzing the VNIS spectra [41]. This discovery provides direct evidence for explaining the composition of the lunar mantle, which has been unclear until now. These results will also improve the models on the formation and evolution of the moon. Lin et al. reported that the spectra of the observed area showed maximum absorption at 1 μm and 2 μm, which can more reliably determine the mineral composition. The analysis shows that the rock in the region is olivine–norite [42].

### 4.3. Application Effects of LMS on Board Chang’e-5

The main scientific mission of the Chang’e-5 mission is to perform lunar sampling return. Therefore, there is no rover similar to the Chang’e-3 and Chang’e-4 missions. The LMS is installed on the Chang’e-5 lander to detect the spectra of the sampling area. The detected spectral data can contribute to the survey of the lunar surface material composition and resources in the sampling area, and to provide scientific data for comparative research on sample laboratory measurements.

**Figure 7:**
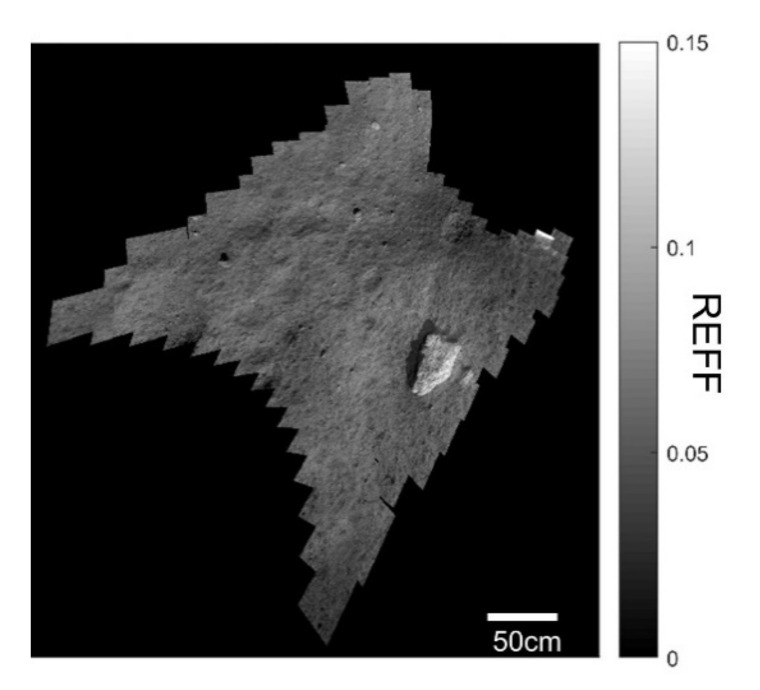
REFF image from the LMS at 900 nm using the full-view mode.

The LMS conducted lunar observations from 21:08 1 December 2020 (UTC) until 17:04 2 December 2020 (UTC), after the Chang’e-5 lander-ascender combination successful soft landing. First, a multi-spectral full-view mode observation was conducted to obtain a wide view of the lunar sampling area, and a six-band panoramic spectral image was acquired in the VIS channel and 14-band spectra in the IR channels (i.e., NIR, SWIR, and MWIR). The REFF image of the sampling area is shown in Figure 7. The spatial resolution of the image was 0.4–1 mm.

During the intervals of sampling transfer by the robotic arm, the LMS conducted full-band observations of the significant targets, sampling targets, and candidate points, and performed in-flight calibration.

## 5. Conclusions

In situ spectral analysis can investigate the surface material composition and weathering conditions of extraterrestrial objects without destroying the original environment. It also provides a unique perspective of data for the study of extraterrestrial objects. Payloads in the form of AOTF spectrometers have the advantages of small volume, low mass, and high spatial adaptability, which are very suitable for in situ spectral detection at close range on the lunar surface. Three AOTF spectrometers have been successfully applied to lunar in situ spectral measurement, including the VNIS on board the Chinese Chang’e-3/4 missions and the LMS on board the Chang’e-5 mission. These instruments have overcome harsh and low light environment and wide temperature variations on the lunar surface, and have successfully achieved spectral acquisition and in situ calibration of lunar objects. These results provide a unique perspective on scientific data for analyzing the mineral composition and evolutionary history of the Moon. This paper discusses three in situ spectrometers successfully applied to the lunar surface and their functionalities, performance parameters of the installed AOTF crystals, data acquisition, and scientific applications of the data. The results can provide reference for the design and application of in situ AOTF spectroscopic instruments applied to extraterrestrial bodies in the future.

## Figures and Tables

**Table 1:** Main performance parameters of VNIS and LMS.

Parameters	VNIS/Chang’e-3		VNIS/Chang’e-4		LMS/Chang’e-5	
	VIS-NIR	SWIR	VIS-NIR	SWIR	VIS-NIR	IR
Spectral coverage/nm	449–950	900–2400	450–950	900–2400	480–1450	1400–3200
Spectral resolution/nm	2–7	3–12	2.4–6.5	3.6–9.5	2.4–9.4	7.6–24.9
FOV/deg	8.5 × 8.5	ø3.6	8.5 × 8.5	ø3.6	4.17 × 4.17	4.17 × 4.17
Effective pixels	256 × 256	1	256 × 256	1	256 × 256	1
Quantization/bits	10	16	10	16	10	16
SNR/dB	≥31@ albedo is 9% and solar incident angle is 45°	≥32@ albedo is 9% and solar incident angle is 75°	≥33@ albedo is 9% and solar incident angle is 45°	≥31@ albedo is 9% and solar incident angle is 75°	≥34@ albedo is 9% and solar incident angle is 45°	≥39@ albedo is 9% and solar incident angle is 45°
Sampling interval/nm	5		5		5	
Power/w	19.8		16.95		15.17	
Weight/kg	4.675/probe ~0.7/electronics		4.675/probe ~0.7/electronics		≤5.57	
Operating temperture	−20 °C~+55 °C		−20 °C~+55 °C		−25 °C~+65 °C	

**Table 2:** Main performance parameters of VNIS and LMS.

Parameters	VNIS/Chang’e-3		VNIS/Chang’e-4		LMS/Chang’e-5	
	VIS-NIR	SWIR	VIS-NIR	SWIR	VIS-NIR	IR
Material	TeO_2_	TeO_2_	TeO_2_	TeO_2_	TeO_2_	TeO_2_
Spectral coverage/nm	449–950	899–2402	450–950	900–2400	480–1450	1400–3200
FWHM/nm	2.3–6.3 @ < 630 nm 2.4–6.8 @ > 630 nm	3.1–8.9 @ < 1380 nm 4.4–11.6 @ > 1380 nm	2.0–5.8 @ < 630 nm 2.8–6.4 @ > 630 nm	3.75–8.4 @ < 1380 nm 4.2–9.6 @ > 1380 nm	2.6–9.4 @ < 780 nm 2.4–9.0 @ > 780 nm	7.6–20.8 @ 1400–2300 nm 11.6–24.9 @ 2200–3200 nm
RF/MHz	70.7–178.6	41.9–118.9	71.2–178.7	42.0–118.8	45.2–163.6	27.7–66.2
Angular aperture/°	>7	>8	>7	>8	>7	>3
Diffraction angle/°	>5.6	>7.5	>5.6	>7.5	>5.6	>7
Power/W	~2		~2		~2	

## Data Availability

Not applicable.
